# Supplementary material for: Computational design and engineering of an Escherichia coli strain producing the nonstandard amino acid para-aminophenylalanine
Source: iScience. 2022 Jun 9;25(7):104562. doi: 10.1016/j.isci.2022.104562 (PMC9249619; doi:10.1016/j.isci.2022.104562)
Supplement: Document S1. Figures S1–S5 and Tables S1 and S2 [file mmc1.pdf]

iScience, Volume *25*

Supplemental information

Computational design and engineering of an *Escherichia coli* strain producing the nonstandard amino acid *para*-aminophenylalanine

Ali R. Zomorrodi, Colin Hemez, Pol Arranz-Gibert, Terrence Wu, Farren J. Isaacs, and Daniel Segrè

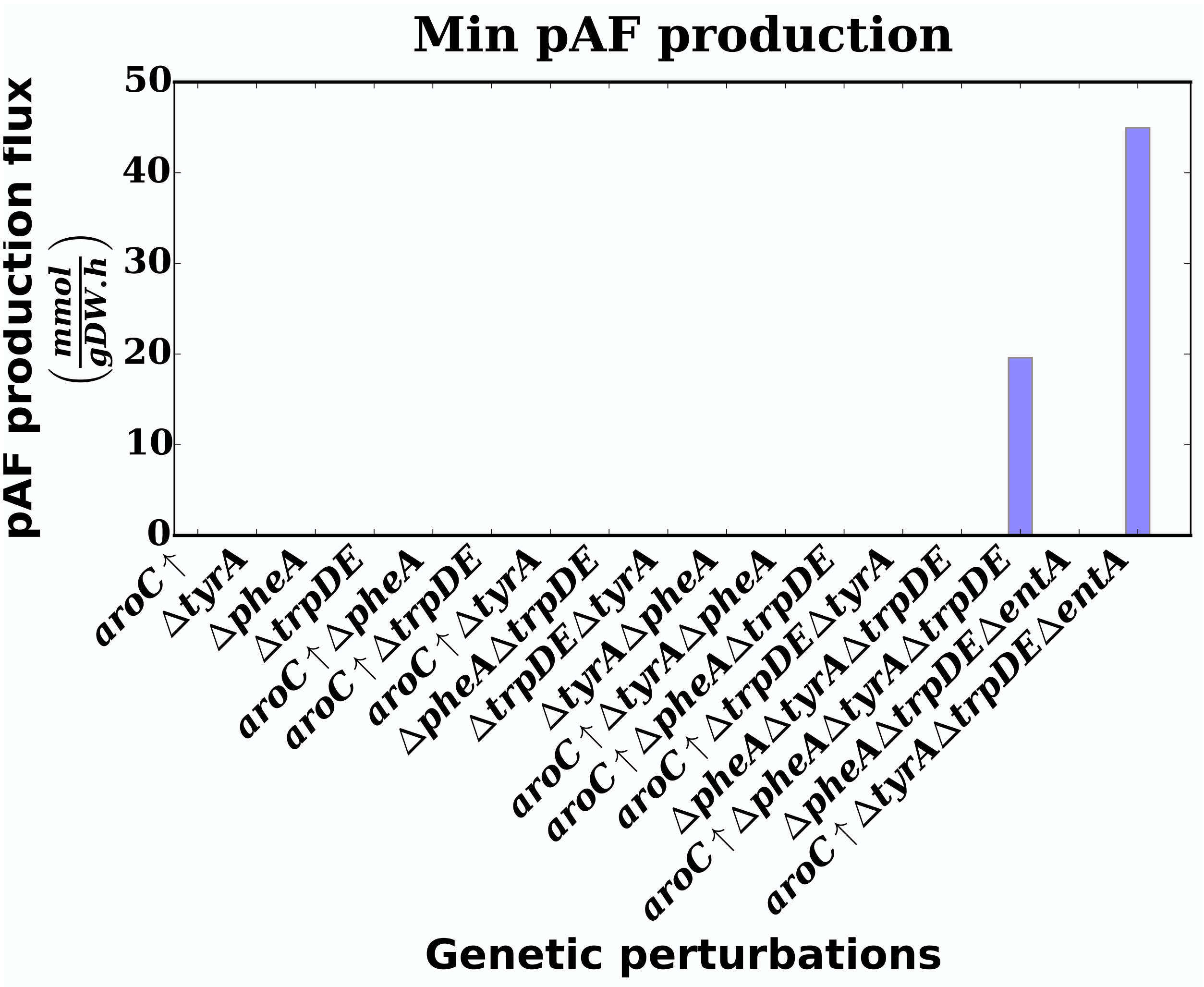

Figure S1. Epistatic interactions among computationally identified genetic manipulations with respect to pAF production as predicted by GEMs of metabolism (gene knockouts implemented for *pheA*, *tyrA*, and *trpDE* instead of downregulations to more closely simulate our experiments). Related to Figure 2 and Results (main text).

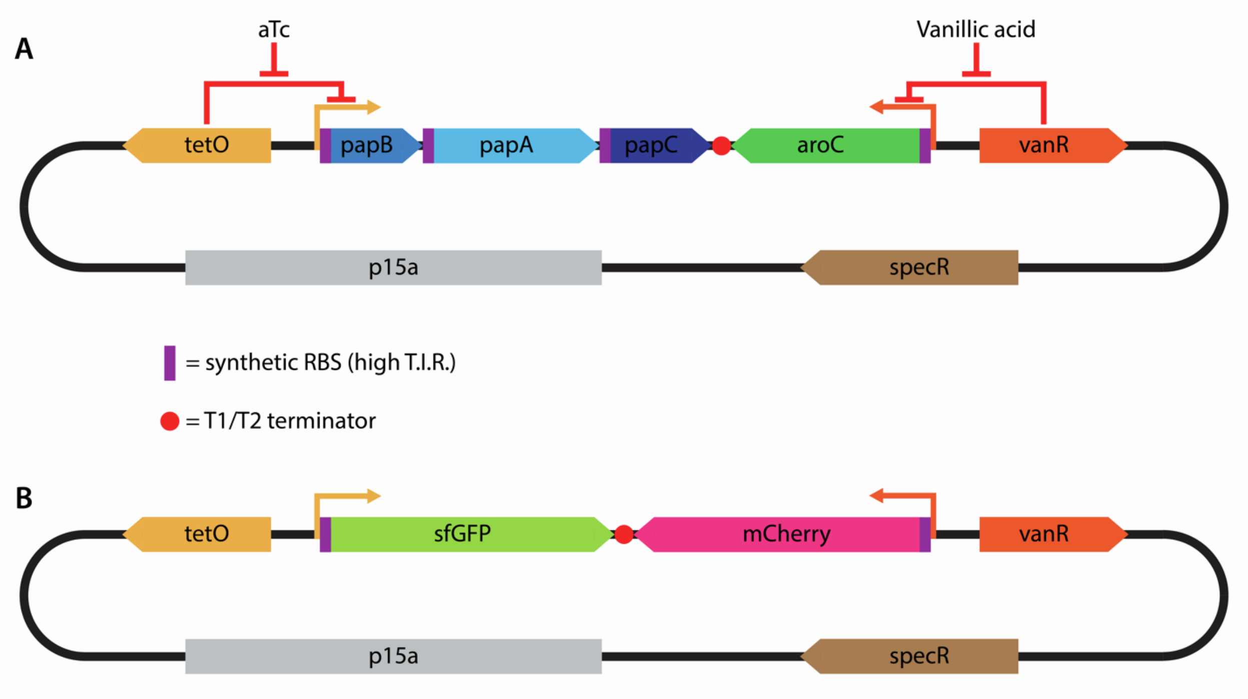

Figure S2. Overexpression of (A) papBAC and aroC or (B) sfGFP and mCherry using dual titratable promoters (T.I.R.: Translation Initiation Rate). Related to Figure 3

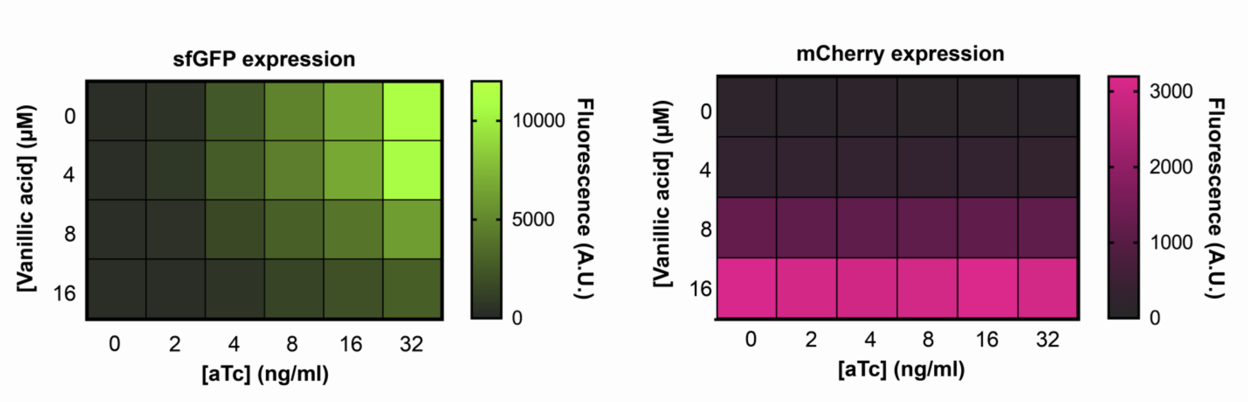

Figure S3. GFP and RFP expression under the dual aTc- and vanillic acid-inducible promoter circuit. Related to Figure 3.

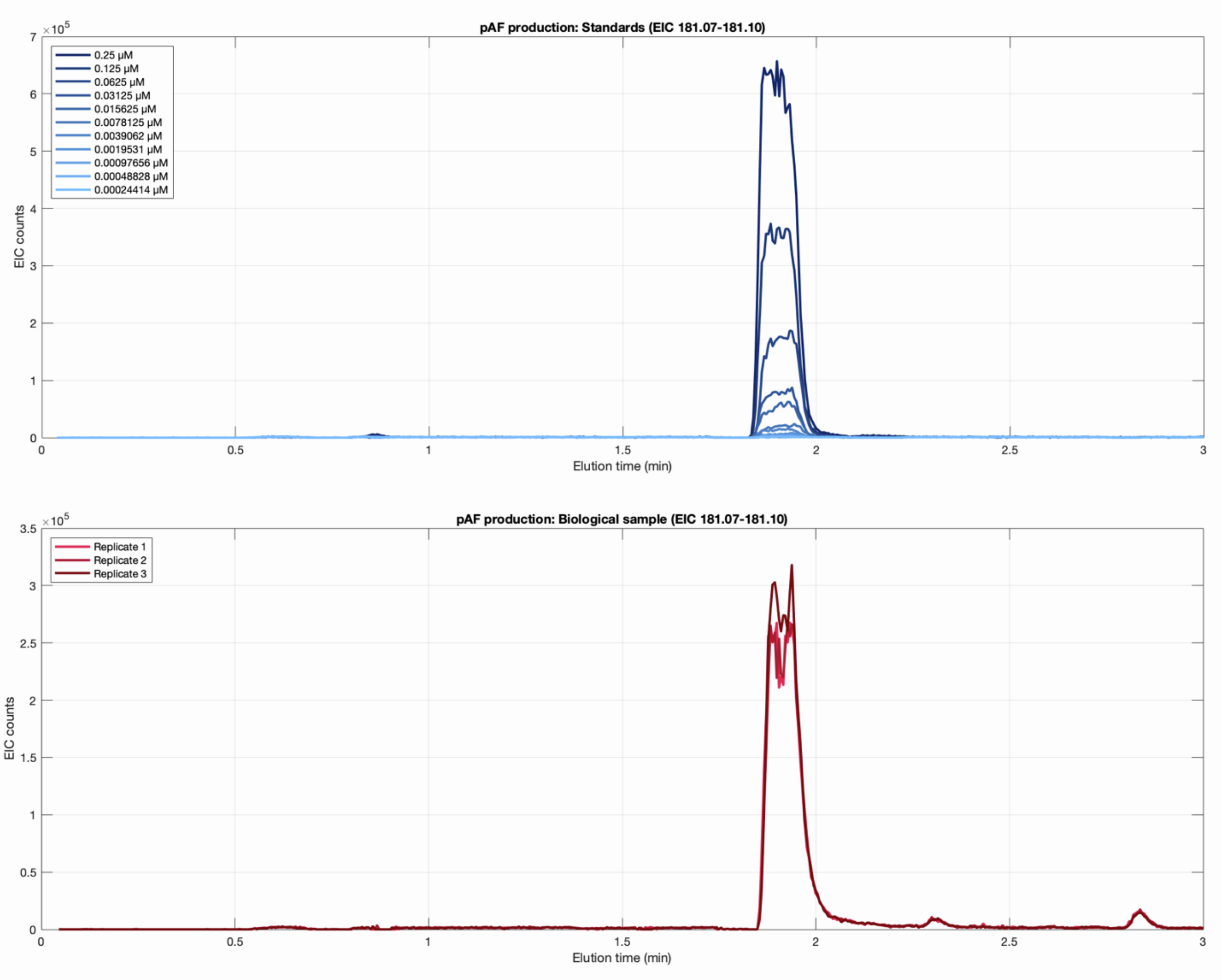

Figure S4. Detection of pAF by LC/MS (top panel) using reference standards generated by adding pAF at known concentrations to the supernatant of non-pAF producing *E. coli* cultures and (bottom panel) from the supernatant of pAF-producing *E. coli* cultures (data shown are positive ion extracted ion chromatograms). Related to STAR METHODS.

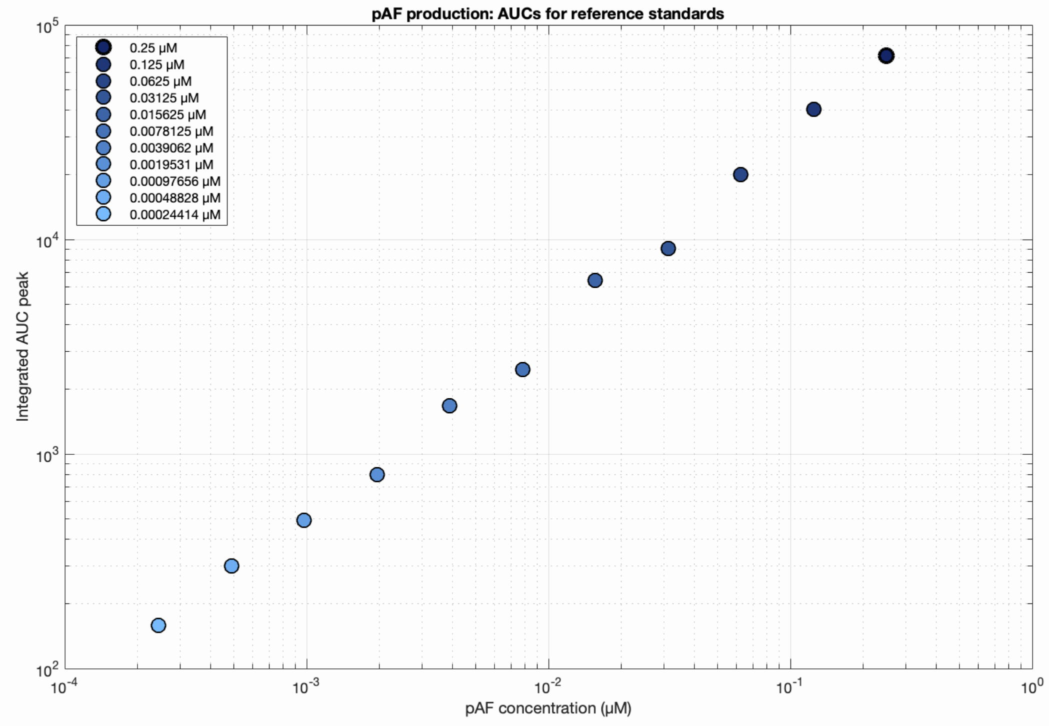

Figure S5. Standard curve generated for pAF quantification by LC/MS. Related to STAR METHODS.

Table S1. Numerical data for the graph shown in the main text Figure 3C. Related to Figure 3.

Strain	Plasmid	Measured doubling time (min)	Relative doubling time	[pAF] (µM)
ECNR2.ΔpheA	pPAF-v5	149 ± 1	1.084 ± 0.016	3.72 ± 0.32
ECNR2.ΔtrpDE	pPAF-v5	143 ±0.02	1.048 ± 0.007	3.3 ± 1.1
ECNR2.ΔtyrA	pPAF-v5	151 ± 0.4	1.099 ± 0.010	2.2 ± 1.0
ECNR2.ΔpheA.ΔtrpDE	pPAF-v5	144 ± 5	1.052 ± 0.041	2.32 ± 0.17
ECNR2.ΔtrpDE.ΔtyrA	pPAF-v5	165 ± 2	1.203 ± 0.023	n.d.
ECNR2.ΔtyrA.ΔpheA	pPAF-v5	153 ± 2	1.116 ± 0.022	n.d.
ECNR2.ΔpheA.ΔtrpDE.ΔtyrA	pPAF-v5	150 ± 1	1.093 ± 0.015	1.83 ± 0.19
ECNR2.ΔpheA.ΔtrpDE.ΔtyrA.ΔentA	pPAF-v5	153 ± 0.6	1.113 ± 0.012	n.d.
ECNR2	pPAF-v5	137 ± 1	1.000 ± 0.014	3.95 ± 0.08
ECNR2.aroFG-FBR	pPAF-v5	124 ± 5	0.907 ± 0.044	6.4 ± 2.2
ECNR2.ΔtyrR	pPAF-v5	157 ± 3	1.145 ± 0.030	n.d.
ECNR2.ΔtyrR.aroFG-FBR	pPAF-v5	161 ± 5	1.175 ± 0.043	36 ± 4.1
ECNR2	pGFP-RFP	142 ± 2	1.038 ± 0.019	n.d.

Table S2. Vitamin and mineral supplements used in all culture conditions. Related to STAR METHODS.

100x vitamin solution (for 500 ml in water)		
**Component**	**Concentration (M)**	**Volume (ml)**
Thiamine HCl	0.02	25
Calcium pantothenate	0.02	25
p-aminobenzoic acid	0.02	25
p-hydroxybenzoic acid	0.02	25
2,3-dihydroxybenzoic acid	0.02	25
Water		375
**50,000x trace metal mix (for 50 ml in water)**		
**Component**	**Formula**	**Amount (g)**
Ammonium molybdate	$(NH_4)_6Mo_7O_{24} \bullet 4H_2O$	0.009
Boric acid	H_3BO_3	0.062
Cobalt chloride	$CoCl_2$	0.018
Cupric sulfate	$CuSO_4$	0.006
Manganese chloride	$MnCl_2$	0.04
Zinc sulfate	$ZnSO_4$	0.007
